# Investigation of beetroot (*Beta Vulgaris*) extracts compared to staurosporine in human breast cancer cells: cellular functions *in-vitro* study

**DOI:** 10.3389/fonc.2026.1740274

**Published:** 2026-01-28

**Authors:** Suzan A. Abushal, Rokayya Sami, Nashi K. Alqahtani, Safa H. Qahl, Ahlam A. Harasani, Nouf A. Babteen, Hend F. Alharbi, Fahad Eid Albalawi, Fatimah Amer, Hajer A. Alfarteesh, Hayat A. Alghamdi, Sara M. Almutairi

**Affiliations:** 1 Program of Food Sciences and Nutrition, Turabah University College, Taif University, Taif, Saudi Arabia; 2 Department of Food Science and Nutrition, College of Sciences, Taif University, Taif, Saudi Arabia; 3 Department of Food and Nutrition Sciences, College of Agricultural and Food Sciences, King Faisal University, Al-Ahsa, Saudi Arabia; 4 Department of Biological Sciences, College of Science, University of Jeddah, Jeddah, Saudi Arabia; 5 Department of Food Science and Human Nutrition, College of Agriculture and Food, Qassim University, Buraydah, Saudi Arabia; 6 College of Medicine, Fahad Bin Sultan University, Tabuk, Saudi Arabia; 7 Department of Biology, College of Science, King Khalid University, Abha, Saudi Arabia; 8 University Medical Clinics, Taif University, Taif, Saudi Arabia

**Keywords:** anticancer agents, apoptosis, cytotoxicity, MCF-7 cells, natural, oxidative stress, red beetroot

## Abstract

The cytotoxic and apoptotic effects of *Beta Vulgaris* (red beetroot) extracts prepared with various solvents, including water, EtOH (ethanol), MeOH (methanol), Ace (acetone), EtOAc (ethyl acetate), and BuOH (butanol), on staurosporine (Sts) in MCF-7 cells have been examined in the present study. The MTT assay showed that cell survival was significantly decreased in the treatment groups, with the extracts of BuOH (30.11%) and Ace (41.17%) showing the highest suppression in comparison to the control. A decrease in viable cells was verified by Trypan blue analysis; the viability of BuOH and Ace extracts was 48.41% and 52.45%, respectively. While cytochrome c release reached 11.08 ng/mL in BuOH-treated cells compared to 1.32 ng/mL in the control, caspase-3/7 activity increased dramatically, reaching 61.48% with BuOH and 53.72% with Ace. Reactive oxygen species (ROS) and hydrogen peroxide (H_2_O_2_) levels were high (62.71% and 5.11 µM, respectively), resulting in a 48.92% reduction in adenosine triphosphate (ATP). Annexin V-FITC/PI staining demonstrated that BuOH and Ace raised the values of early and late apoptosis by 26.19% and 29.47%, respectively. The BuOH extract had the strongest cytotoxic and pro-apoptotic effects across most assays, with the lowest survival rate (30.11%), high caspase-3/7 (61.48%), high cytochrome c release (11.08 ng/mL), strong ROS (62.71%), and H_2_O_2_ production (5.11 µM), and the highest early and late apoptosis rates (26.19% and 29.47%). The findings demonstrate the potential of *Beta Vulgaris* chemical compounds as natural anticancer agents against breast cancer cells.

## Introduction

Cancer is categorized as a chronic, non-communicable disease and is one of the most serious health issues. Despite a wealth of oncotherapy research and the rapid advancement of novel diagnostic and treatment techniques. Numerous cancer forms continue to rise in incidence. Despite the fact that living conditions are getting better, poor lifestyle choices, such as an unbalanced diet or stress exposure, are considered main causes (1). Other genetic and environmental risk factors have been identified, including smoking, excessive alcohol use, inflammatory bowel disease, eating a lot of red and processed meat, obesity, and diabetes (2). Chemotherapy is one of the main cancer treatments, along with surgery and radiation. Chemical drugs cause cell death by inducing an apoptotic pathway and acting on cancer cells at various stages. However, traditional chemotherapeutic medicines frequently target cancer cells that divide quickly as well as healthy cells, which can cause negative effects for patients (3). The absence of completely effective therapy remains a challenge for modern medicine even with advancements in treatment and the application of novel chemotherapeutics. Thus, there is a great need to apply the knowledge to create and synthesize novel, promising chemotherapeutics that would be effective against cancer cells, as well as to appropriately highlight prevention, such as adopting healthy eating habits that include consuming a lot of fruits and vegetables, which have been shown to lower the risk of developing cancer. Maintaining a healthy weight and engaging in regular physical activity can help prevent cancer cases (4).

**Figure 1:**
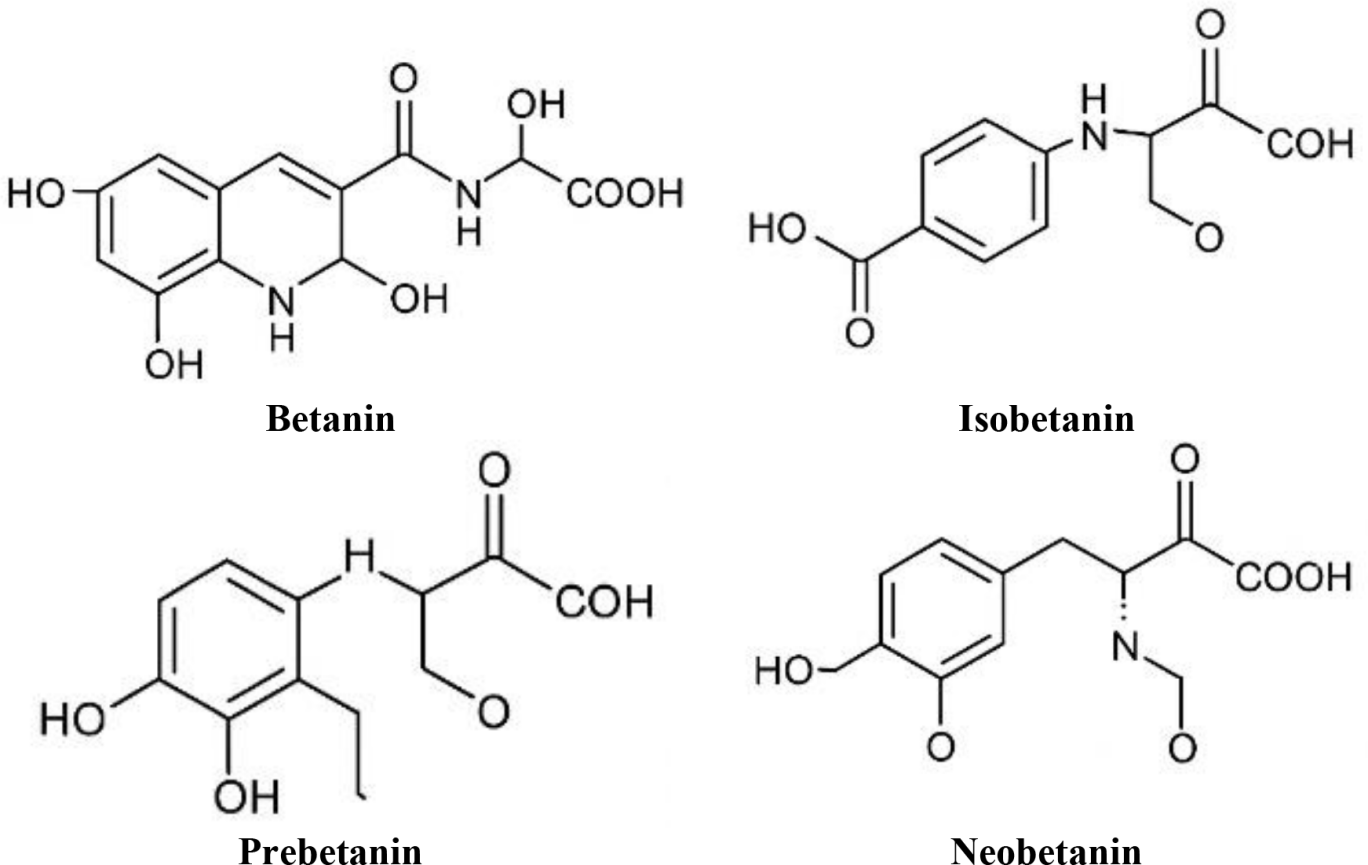
The chemical structure of the main pigments in beet root.

New dietary components high in antioxidants and other metabolically active compounds could serve as a basis for such preventative actions. It is still difficult to find components that have both low cytotoxicity to healthy cells and anticancer impacts. With more than 60% of currently used anticancer medicines coming from natural sources, several chemicals derived from plants have demonstrated anticancer properties and have drawn interest in the prevention and treatment of cancer (5). The urgent need to support non-pharmacological approaches for treating diseases, including cancer, has led to an increase in interest in plant-based raw materials, particularly “folk” plants that may be effectively employed in prevention. Beetroot (*Beta Vulgaris*), a vegetable which belongs to the *Chenopodiaceae* family, is widely cultivated, particularly in many countries. It has high antioxidant activity and a more abundant supply of total polyphenols with anti-carcinogenic potentials (6). The type of extremely readily available natural antioxidant pigments known as betalains, which represent between 75%: 90% of the total pigments in red beets. The other pigments include (betanin, isobetanin, prebetanin, and neobetanin). **Figure 1** presents the main pigments in beetroot.

Its high concentration of bioactive components, such as flavonoids, carotenoids, polyphenols, and betalains, provides significant nutritional and health benefits (7). Numerous health benefits, including antidepressant, antioxidative stress, hematopoietic, anti-inflammatory, antibacterial, anti-proliferative, diuretic, hepatoprotective, anti-nephrotoxic, immunomodulatory, antihypercholesterolemic, antihyperglycemic, and anti-carcinogenic activities, have been identified in *in-vitro* and preclinical studies regarding beetroot. Consuming beetroot may also help in the prevention of age-related diseases. Since beetroot contains a lot of inorganic nitrate and betaine, it has an impact on the blood vessels’ vascular endothelium that enhances blood flow (8).

Beetroot was utilized to improve running performance and workout stamina. Accordingly, it may have antidiabetic and hepatoprotective properties. However, under a variety of circumstances, including temperature, pH, light, and oxygen, betalains and phenolic compounds are prone to quick breakdown. Consequently, the low stability of these chemicals enables processing beetroot extract extremely difficult (9).

Since each extraction liquid has a different polarity and extraction effectiveness, it is essential to use a variety of these substances while researching the effects of beetroot on breast cancer cells. These liquids include water, ethanol, methanol, acetone, ethyl acetate, and butanol. Hydrophilic substances such as betalains and phenolic acids, which are recognized for their high antioxidant and possible anticancer qualities, are best extracted using polar solvents such as water, ethanol, and methanol. On the other hand, certain bioactive compounds, such as flavonoids and other non-polar pigments, can be extracted using moderately polar or less polar solvents including acetone, ethyl acetate, and butanol. These solvents may also have cytotoxic or apoptosis-inducing effects on breast cancer cells. Researchers can ascertain which extraction liquid produces the strongest anticancer chemicals and have a better knowledge of beetroot’s therapeutic potential against breast cancer by comparing the biological activity of extracts obtained with these various solvents. It has been shown that consumption of 40 mM betanin to the human chronic myeloid leukemia cell line K562 reduces cell growth by 50% and initiates an intrinsic apoptotic pathway by activating caspase-3, an executioner caspase in apoptotic cascades; additionally, beetroot has been used as a functional food and medicinal product by individuals suffering from breast, colon, and prostate cancer (10). According to *in-vitro* research, beetroot extract can have a cytotoxic effect on both androgen-independent human prostate cancer cells (PC-3) and estrogen receptor-positive human breast cancer cells (MCF-7). Furthermore, when beetroot extract and the chemotherapeutic medication doxorubicin were administered to cancer cells at the same time, a synergistic impact was observed (11). Furthermore, by promoting the production of detoxifying and antioxidant enzymes and lowering oxidative stress caused by xenobiotics, betanin may be crucial in preventing cancer, liver, lung, and kidney damage (12). Previous research suggests that red beetroot, betanin, its primary ingredient, and other substances with potent antioxidant, antiproliferative, and anticancer properties may be useful in the early stages of cancer development and progression (13).

Investigating the effects of beetroot extracts on MCF-7 cells using various extraction liquids (water, EtOH (ethanol), MeOH (methanol), Ace (acetone), EtOAc (ethyl acetate), and BuOH (butanol)) and comparing their activity with staurosporine (Sts) in controlling cellular functions was the scope of this study. By comparing and analyzing the biological activity of different extracts, the study aimed to identify the best extraction solvent and show beetroot’s potential as a natural source of chemotherapy medication.

## Materials and methods

### Sample preparation

Fresh red beetroot samples were obtained from a local market in Taif, Saudi Arabia, and prepared for laboratory analysis using a standard drying and processing procedure. Roots were properly cleansed with distilled water, peeled, and cut into small pieces before being dried at 40 °C in a hot air oven (Yiheng, Shanghai Ins. Co., Ltd., China) until their weight maintained constant. A laboratory grinder (MF-10, Jingxin Co., Ltd., China) was used to grind the dried material into a fine powder. 100 mL of various solvents, including distilled water, 70% ethanol, 80% methanol, 70% acetone, pure ethyl acetate, and butanol, were used to soak 10 grams of beetroot powder. For better pigment degradation, each mixture was macerated for 24 hours in the dark at room temperature (25 °C) while being continuously shaken. The extracts were centrifuged at 2,500 rpm for 15 minutes (14). Following extraction, solid residues were eliminated from the combinations by squeezing and filtering through Whatman No. 1 filter paper. A rotary evaporator (YRE 2000E, Gongyi, China) was used to concentrate the filtrates at 50 °C to dryness under decreased pressure. The extracts were then dried in a desiccator to eliminate any leftover solvent. The dried extracts were weighed, noted, stored in airtight containers, and kept at -20 °C until additional biological investigations.

### Cell culture

Cell culture human epithelial cell lines for breast cancer (MCF-7) were obtained from the Shanghai Institute of Biological Sciences in China. Tissue culture flasks containing DMEM supplemented with 100 units/mL of penicillin-streptomycin, 0.14% sodium bicarbonate, 0.1 mM sodium pyruvate, and 100 mg/mL streptomycin supplemented with 10% heat-inactivated Fetal Bovine Serum (FBS) (Sigma-Aldrich, Louis, USA) were used to cultivate these cells at 37 °C and 5% CO_2_ incubators (WJ 3T series, Shanghai, China) under humidified conditions (85%) until the bilayer of cell density was achieved (20). These cells (3 × 10^4^ cells mL^-1^) were cultured for 48 hours after being regularly plated, examined, and confirmed. The investigations were conducted on cells that were in the exponential development phase (70 - 80% confluency). Cells were incubated for five minutes prior to being mixed with 15 mL of complete media. Following a 10-minute centrifugation of the cell solution at 3,500 rpm, the recovered cells were utilized for *in-vitro* study.

### Beetroot extract preparation for breast cancer cell lines

The dried beetroot extracts were reconstituted in dimethyl sulfoxide (DMSO). For achieving a stock concentration of 10 mg/mL, each extract was dissolved. The sterility of the stock solutions was ensured by vortexing completely and filtering through 0.22 µm syringe filters. Final working concentrations ranging from 25 to 400 µg/mL were obtained by diluting the stock solutions in DMEM supplemented with 10% FBS and 1% penicillin-streptomycin. The ultimate DMSO concentration in every treatment well was kept below 0.5% to prevent solvent-induced cytotoxicity. All tests included vehicle controls with comparable DMSO doses devoid of extract to guarantee precise interpretation of the cytotoxic effects.

### Staurosporine preparation as a positive control for breast cancer cell lines

**Figure 2:**
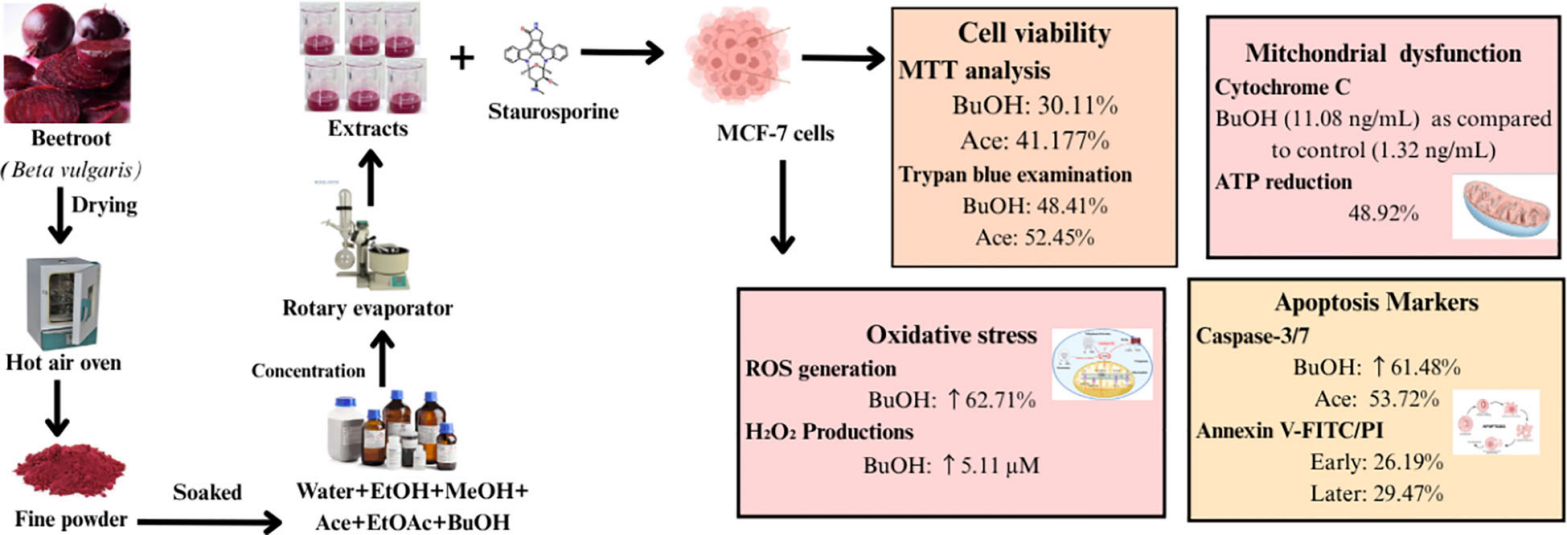
Summary of beetroot extracts and staurosporine experimental work on d MCF-7 human epithelial cell lines.

In contrast to the effects of plant extracts, staurosporine has been used as a positive control to confirm the activation of apoptosis in MCF-7 cells. Staurosporine was purchased from Sigma-Aldrich (62996-74-1, St. Louis, Missouri, USA), and it was dissolved in DMSO to create a 1 mM stock solution. The stock was diluted in culture medium just before use to decrease the working concentration below 1 µM. The cells were incubated for 24 hours at 37 °C with 5% CO_2_ while being treated with staurosporine. A baseline comparison was made using untreated cells and solvent controls (DMSO at final concentrations ≤ 0.1%). **Figure 2** presents the summary of beetroot extracts and staurosporine on MCF-7 human epithelial cell lines.

### Cytotoxicity

Cell viability as a measure of cytotoxicity has been measured using the methyl thiazol tetrazolium (MTT) technique. In order to promote cell adhesion, MCF-7 cells were planted in 96-well plates (10^4^ cells/well) and incubated for the whole night at 37 °C with 5% CO_2_. The cells were exposed to extracts of 200 µg/ml red beetroot extracts, as well as staurosporine as a positive control. Following a 24-hour treatment period, each well received 20 µL of MTT reagent (5 mg/mL) in phosphate buffered saline (PBS) and was incubated for four hours. Following the removal of the medium, 100 µL of DMSO was added in order to dissolve the formazan crystals. A microplate reader (RT 2100C, Shenzhen, China) was used to measure absorbance at 570 nm after the plates were shaken for 10 minutes and left in the dark at room temperature for a whole night. The negative control consisted of untreated cells (10). The cell survival rate, which is based on the percentage of absorbance difference between the experimental group and the blank control group, was used to determine the growth inhibitory effects of the supernatants.

### Trypan blue exclusion

The ability to survive was determined using the Trypan blue exclusion procedure, which separates living cells from dead cells according to membrane integrity. In order to promote adhesion, MCF-7 cells were seeded in 6-well plates (1 × 10^2^ cells/well) and incubated for the whole night at 37 °C with 5% CO_2_. The cells were exposed to various extracts of *Beta Vulgaris*, as well as staurosporine as a positive control. Trypsinization was used to gather adherent and floating cells after a 24-hour centrifugation for five minutes at 1,000 rpm was applied. After resuspending the cell in PBS, 10 µL of 0.4% Trypan blue dye was combined with 20 µL of the cell suspension. The mixture was placed onto a hemocytometer (BK-CC10, Shandong, China) and examined using an automated cell counter (C100-SE, Shenzhen, China) following a two-minute incubation process at room temperature. Unstained cells were regarded as viable, whereas blue-stained cells were classified as non-viable (15).

### Caspase-3/7 activity

The apoptotic effects of *Beta Vulgaris* extracts on MCF-7 cells were examined using caspase-3 and -7 activities, which are important markers of apoptosis, with staurosporine acting as a positive control. The cells were incubated for the whole night at 37 °C with 5% CO_2_ after being seeded in 96-well plates with 1 × 10^4^ cells per well. Following a 24-hour exposure to the extracts, cell lysates were prepared using a Caspase-3/7 Kit (Sigma-Aldrich, Ac-DEVD-AMC, USA) in compliance with the manufacturer’s instructions. Caspase-3/7 reagent and cell lysate were added in equal amounts to each well, gently mixed, and allowed to remain at room temperature in the dark for an hour (16). For evaluating apoptosis in comparison to untreated control cells, a microplate luminometer (CLIA, Yantai, China) was used to quantify luminescence, which is proportional to caspase-3/7 activity.

### Lactate dehydrogenase release

The cytotoxicity and membrane integrity of MCF-7 cells treated with staurosporine and extracts from *Beta Vulgaris* were evaluated using the LDH release assay. In 96-well plates (1 × 10^4^ cells/well), the cells were seeded and incubated at 37 °C with 5% CO_2_ during the whole night. The culture mediums were washed twice in ice-cold PBS, gathered, and centrifuged for five minutes at 4,000 rpm at 25 °C following a 24-hour treatment with increasing extract concentrations (10 - 100 µg/mL). The cytotoxicity detection LDH was used to find out that lactate was being converted to pyruvate according to the protocol of the LDH Kit (Sigma-Aldrich, TOX7, USA). 50 µL of the LDH reaction mixture kit was combined with 50 µL of the supernatant from each sample on a fresh 96-well plate. A microplate reader was used to detect optical densities of red color at 490 nm after the plate was incubated for 30 minutes at 25 °C in the dark (17). The cells that received the highest LDH release control were treated with 1% Triton X-100.

### Reactive oxygen species

The intracellular ROS levels in MCF-7 cells treated with staurosporine and *Beta Vulgaris* extracts were measured by using the 2′,7′-dichlorofluorescein diacetate (DCFH-DA) assay. 96-well black-walled plates were seeded with 1 × 10^4^ cells per well, and the cells were incubated at 37 °C with 5% CO_2_ throughout the whole night. Following a 24-hour treatment period, cells were rinsed with PBS and incubated for 30 minutes at 37 °C in the dark with 10 µM DCFH-DA kit (35845, Sigma-Aldrich). After removing any extra dye, PBS was used to wash the cells once again. A microplate reader was used to measure fluorescence intensity at 485/538 nm filter (18). Relative ROS in percentage with comparison to the untreated control cells was used to express the results.

### Adenosine triphosphate

The ATP kit (CS224 series, Luminescent, Sigma, USA) was used to measure intracellular ATP levels as a measure of metabolically active MCF-7 cells treated with staurosporine and extracts from *Beta Vulgaris*. Following a 24-hour treatment, cells were resuspended in 50 µL of new culture media to maintain appropriate buffer conditions, and 50 µL of ATP reagent and 50 µL of 1X PBS were added. Following providing the mixture a slight shake by a plate shaker (SK-L330-Pro, Beijing, China) and covering it to keep out light, it was incubated at 25 °C for 30 minutes (15). A microplate reader was then used to detect luminescence, and the signal strength was shown to be associated with the quantity of ATP and expressed as a percentage.

### Bradford protein

Total protein levels in MCF-7 cells were measured using the Bradford protein assay as an indirect measure of metabolic activity. Cells were lysed in ice-cold Radioimmunoprecipitation (RIPA) buffer containing a protease inhibitor cocktail after being treated for 24 hours. Cells were cleaned with cold PBS. The Bradford protein assay was applied by following the manufacturer’s instructions for the bicinchoninic acid (BCA) method. 50 µL of BCA (Reagent A) and 50 µL of BCA (Reagent B) were mixed (19). After centrifuging the lysates for 15 minutes at 4 °C at 10,000 rpm, the supernatants were gathered for examination. In order to quantify proteins, 10 µL of each sample was combined with 200 µL of diluted Coomassie Brilliant Blue reagent (B6916, Sigma-Aldrich, USA) in a 96-well plate. The mixture was incubated at 37 °C for 30 minutes. A microplate reader was used to assess the absorbance at 595 nm in comparison to bovine serum albumin (BSA) standard (20).

### Hydrogen peroxide production

The Amplex Red Hydrogen Peroxide/Peroxidase Assay kit (MAK165-1KT, Sigma-Aldrich, USA) was used for evaluating hydrogen peroxide (H_2_O_2_) generation in accordance with the manufacturer’s instructions. MCF-7 cells were seeded at a density of 1 × 10³ cells/well in black 96-well plates and were left to adhere overnight. Cells were exposed to samples for 24 hours. Following treatment, 100 µL of the active reaction mixture containing 50 µM Amplex Red reagent and 0.1 U/mL horseradish peroxidase (HRP) in Krebs-Ringer phosphate buffer was added to the culture medium. The plates were incubated in the dark at 37 °C for 30 minutes. A microplate reader was used to measure the fluorescence intensity (Ex. 530 nm/Em. 590 nm), while results were expressed in µM (21).

### Nitric oxide production

Using the Griess reagent method, nitric oxide (NO) generation was examined in MCF-7 cells treated. After being seeded in 96-well plates (1 × 10^4^ cells/well), the cells were cultured for a whole night at 37 °C with 5% CO_2_. Following a 24-hour treatment period at doses of 25 and 50 µg/mL, 100 µL of each well’s culture supernatant was combined with 100 µL of recently made Griess reagent (Sigma-Aldrich, Germany), which contained 0.1% N-(1-naphthyl) ethylene diamine dihydrochloride and 1% sulfanilamide in 2.5% phosphoric acid. A microplate reader was used to detect absorbance at 450 nm against NaNO_3_ as a standard, and the mixture was incubated for 15 minutes at room temperature in the dark, while results were expressed in µM (17).

### Annexin V-FITC/PI apoptosis

An Annexin V-FITC/PI apoptosis detection kit (APOAF, Sigma-Aldrich, USA) was used to measure the rate of apoptosis in MCF-7 cells after treatment with red beetroot extracts and staurosporine in accordance with the manufacturer’s instructions. Each sample was applied to MCF-7 cells in 6-well plates for 24 hours. Following incubation, both adhering and floating cells were gathered, thrice cleaned with cold PBS, and then resuspended in 100 µL of 1× binding buffer. Each sample was then treated with 5 µL of Annexin V-FITC and 5 µL of propidium iodide (PI). The solutions were allowed to stand at room temperature in the dark for 15 minutes. A microplate reader with particular optical filters appropriate for FITC and PI detection (HEALES MB-580, Shenzhen, China) was used to measure fluorescence. For FITC (Annexin V), the device was calibrated to identify early apoptotic cells at an excitation wavelength of 488 nm and an emission wavelength of 530 nm (22). PI, which represents late apoptotic or necrotic cells, had an excitation wavelength of 535 nm and an emission wavelength of 617 nm. For decreased background fluorescence, all measurements were performed using black 96-well plates at room temperature. Early and late apoptotic cell populations were measured using the recorded fluorescence intensities for FITC and PI compared to untreated control samples. The percentages of early and late apoptotic cells in the population were measured and reported.

### Cytochrome C release

A Human Cytochrome c Quantikine ELISA kit (NADPH, CY0100, Sigma-Aldrich, USA) was used to measure the release of cytochrome c from mitochondria into the cytosol, as a sign of intrinsic apoptosis, in accordance with the manufacturer’s instructions. Samples were applied to MCF-7 cells grown in 6-well plates for 24 hours. In order to separate the cytosolic extract from intact mitochondria, cells were extracted after treatment and lysed using a digitonin-based mitochondrial extraction buffer. For certain equal loading, the BCA assay was used to measure the protein quantities in cytosolic extracts. Each well of a pre-coated 96-well plate was filled with 100 µL of samples and cytochrome c standard. A microplate reader was used to measure absorbance at 450 nm, and cytochrome c concentrations were expressed in ng/mL (23).

### Statistical analysis

All data findings were displayed as means with standard deviation (S.D.) and all experiments were conducted in triplicate. The data was examined using one-way analysis of variance (ANOVA) by Tukey’s Honest Significant Difference (HSD) *post-hoc* test, and SPSS software was statistically analyzed (v.16). While P values between 0.05 and 0.001 have been considered significant.

## Results and discussion

In order to extract a wide range of phytochemicals from Beta vulgaris, different solvents were purposefully chosen for this investigation based on their polarity differences. While semi-polar and non-polar solvents like acetone, ethyl acetate, and butanol preferentially extract less polar constituents like alkaloids, terpenoids, and some lipophilic bioactive molecules, polar solvents as water, ethanol, and methanol are efficient at extracting hydrophilic compounds including phenolics, flavonoids, and betalains. This solvent-based fractionation is frequently employed to assess the effects of solvent polarity on biological activity and phytochemical composition.

### Cytotoxic effects of *Beta vulgaris* extracts on MCF-7 on survival rate

**Figure 3:**
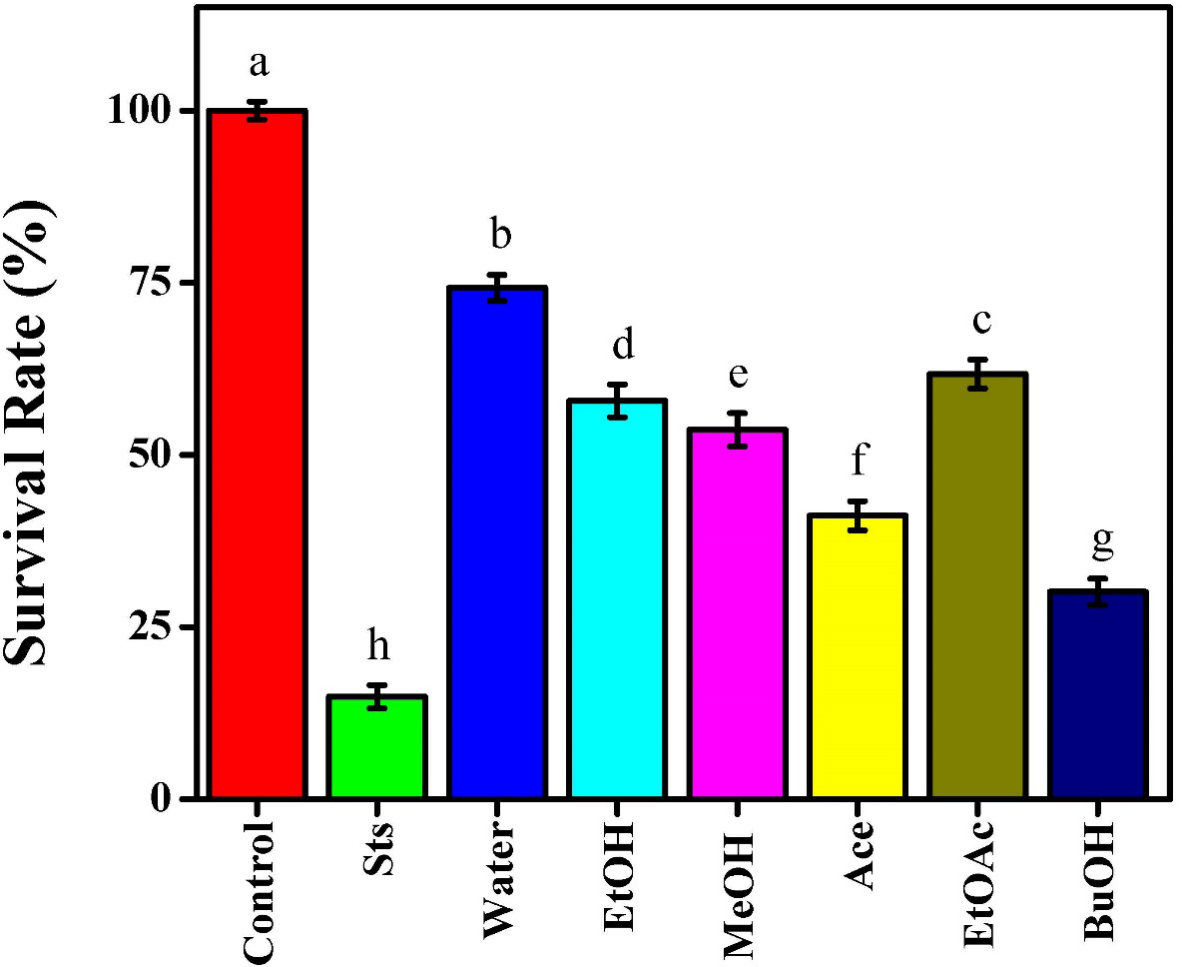
Effect of beetroot extracts and staurosporine on the survival rate of MCF-7 cells; as known Different letters (a, b, c, d, e, f, g, and h) mean significant differences among extracts.

The cytotoxic effects of extracts from *Beta Vulgaris* have been evaluated in a comparison with staurosporine (Sts) as a positive control on MCF-7 cells. The survival rates varied significantly among the solvent extracts, suggesting that the extraction procedure had a major impact on the compound’s bioactivity in beetroot. Sts demonstrated its efficacy in inducing apoptosis by having the strongest cytotoxic effect, reducing cell viability to 14.88%, **Figure 3**. BuOH was the most cytotoxic of the beetroot extracts, lowering cell survival to 30.11%. This suggests that moderately polar solvents may have been used to separate compounds with significant anticancer potential, which are probably abundant in flavonoids and betalain derivatives. Following Ace extract, which also shown a severe cytotoxic impact (41.17%), the survival rates of MeOH and EtOH extracts were 53.64% and 57.85%, respectively. According to these results, polar organic solvents might be used to extract bioactive compounds that could inhibit MCF-7 cell proliferation. The water extract had a higher survival rate (74.25%), indicating less cytotoxic effect. This might be to the presence of some cytotoxic phytochemicals that are non-polar or semi-polar are less soluble in aqueous solutions. EtOAc extract produced an intermediate impact (61.74%), suggesting a modest extraction of active components. Overall, the findings showed that *Beta Vulgaris* extracts had cytotoxic effects and that the effectiveness of the extracted bioactive compounds was influenced by the polarity of the solvent. Beetroot’s potential as a natural source of anticancer drugs is supported by the significant cytotoxic activity seen with BuOH and Ace extracts, which revealed that it contains powerful ingredients capable of influencing the viability of MCF-7 cells. Both flavonoids and phenolic chemicals, which have antioxidant properties, are abundant in beets (14). It has been shown that there is a connection between the development of cancerous cells and endogenous antioxidant mechanisms due to the decreased activity of those mechanisms (24). Additionally, Beck et al. (25), investigated into propolis’ ability to stop Ehrlich Ascites Carcinoma (EAC) cells from growing and spreading in mice. The primary red pigment, betanin, and doxorubicin, anthracycline anticancer drugs, have remarkably similar chemical structures and configurations. The existence of a six-membered sugar molecule and a planar aromatic chromophore. They are especially important since they have been proposed as potential active sites for the intercalation of doxorubicin and its analogs with DNA in cancer cells. This indicates that betanin may play a major role in the reported cytotoxic impact of the red beetroot extract through a possible mechanism of action common with doxorubicin and related anthracycline chemotherapy medications. A different mechanism of chemoprevention by beetroot betacyanins has recently been proposed. It is based on the reduction of inflammation and antagonistic lymphocyte infiltration during the development of esophageal tumors in rats (26). Betacyanins can help in reducing inflammation since they possess potent antioxidants. Betaine (trimethylglycine), one of the other components of beetroot extract, can methylate DNA in cancer cells, potentially acting as a cytotoxic agent (10).

### Effects of *Beta vulgaris* extracts on viable and non-viable carcinogenic cells

**Figure 4:**
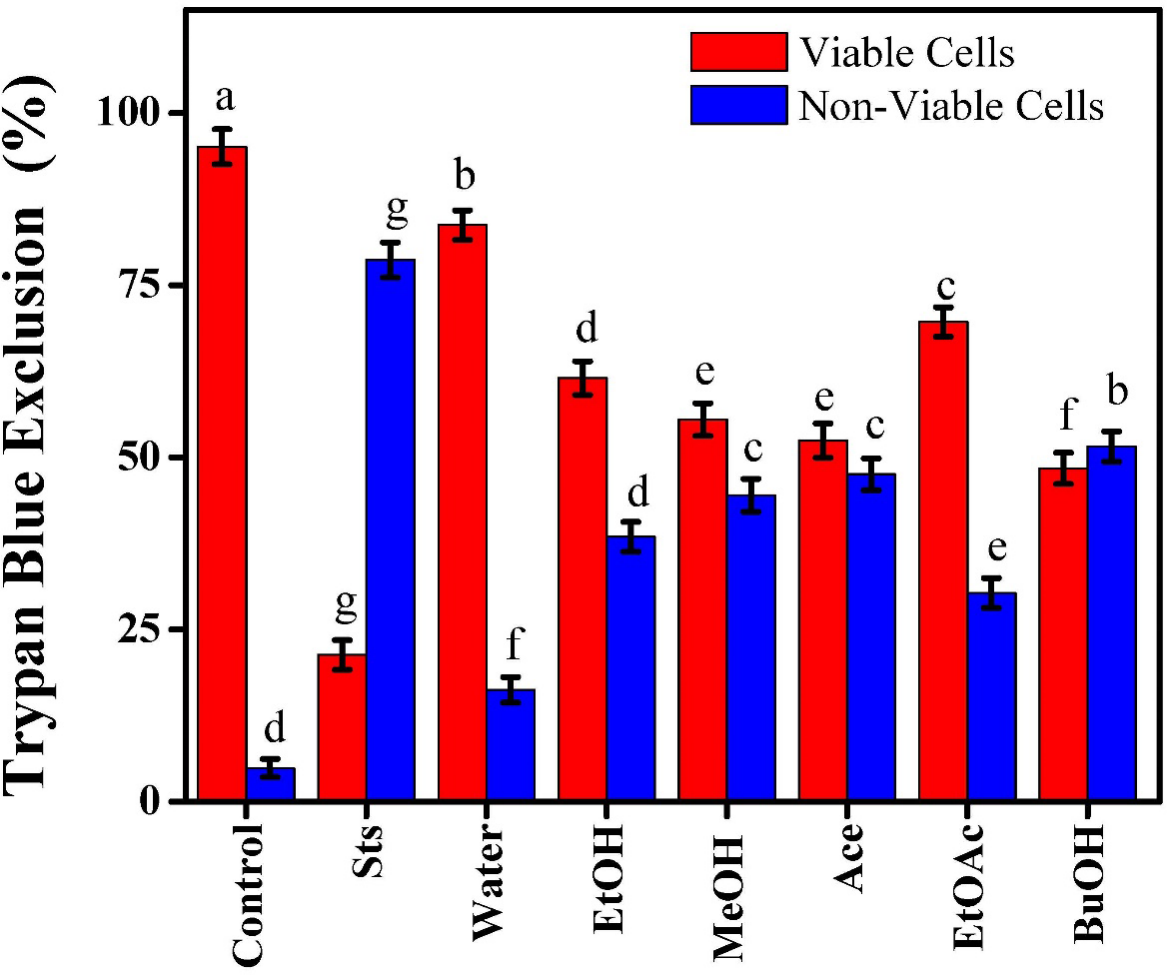
Effect of beetroot extracts and staurosporine on the viable and non-viable carcinogenic MCF-7 cells; as known Different letters (a, b, c, d, e, f, g, and h) mean significant differences among extracts.

The cytotoxic and viability effects of *Beta Vulgaris* extracts on MCF-7 cells were detected using the Trypan blue exclusion test in comparison to the reference apoptotic inducer, Sts. Viable cell percentages clearly decreased after treatment with the different extraction samples of water, EtOH, MeOH, Ace, EtOAc, and BuOH, indicating solvent-dependent variations in cytotoxic activity. The assay’s validity was confirmed by the observation of 21.32% viable cells and 78.68% non-viable cells for Sts, which had the maximum cytotoxic impact as predicted, **Figure 4**. The BuOH sample of beetroot extracts showed the highest cytotoxicity, with 48.41% of viable cells and 51.59% of non-viable cells. This suggests that the semi-polar compounds extracted in butanol were the most effective at damaging cells. The Ace and MeOH samples both shown significant cytotoxic effects, with 52.45% and 55.5% viable cells, respectively. This suggests that these solvents extracted potent bioactive chemicals that may compromise the integrity of cell membranes and trigger apoptosis. The EtOH and EtOAc samples exhibited moderate cytotoxicity, maintaining 61.52% and 69.68% viable cells, respectively, whereas the water extract exhibited the least level of cytotoxic action, retaining 83.75% viable cells and 16.25% non-viable cells. Compounds with more biological activity were naturally extracted by moderately polar solvents, while hydrophilic molecules with weaker anticancer activities were mostly extracted by highly polar solvents such as water.

These findings demonstrated that beetroot extracts prepared with moderately polar solvents, including BuOH, Ace, and MeOH, had greater cytotoxic effects against MCF-7 cells and were consistent with the MTT results. The differences between the extracts showed how solvent polarity influences the yield and structure of phytochemicals such as betalains, flavonoids, and phenolic acids. Therefore, even if their cytotoxic effects were still weaker than those of Sts, the study supported the potential of *Beta Vulgaris* extracts, particularly BuOH and Ace, to be effective natural medicines for the treatment of breast cancer. Proteins and intracellular molecules are frequently released into the surrounding media after cell death, which are obvious indications of membrane integrity breakdown. Additionally, this disruption allows for possible external dyes, such as Trypan blue, to enter the cell and attach to internal structures, enabling the identification of injured and dead cells. Furthermore, because of their ability to stop tumor growth and initiate programmed cell death, plant alkaloids and their derivatives have long been known to play a critical role in anticancer treatments (27). *Beta Vulgaris* contains alkaloids, which may contribute to the plant’s reported anticancer properties. Alkaloids are a broad class of secondary metabolites that include nitrogen and have a variety of pharmacological actions, such as cytotoxic, antioxidant, and antiproliferative qualities. Compared to other phytochemicals as phenolic acids and betalains, alkaloids are present in beetroot in relatively small amounts, but their biological significance should not be neglected (28). It has been demonstrated that beetroot contains nitrogenous compounds equivalent to betaine alkaloids, which are thought to enhance cellular stress responses and promote apoptosis in cancer cells. Alkaloids are known to interact with DNA and disrupt cell division, which stops cancer cells from proliferating. Their ability to modify signaling pathways linked to oxidative stress and apoptosis further supports their significance in initiating programmed cell death. When paired with betalains, strong antioxidants with demonstrated anticancer activity, beetroot’s alkaloid content may intensify the cytotoxic effects on breast cancer cells (29).

### Effects of *Beta vulgaris* extracts on apoptosis, membrane integrity, oxidative stress, and cellular energy in MCF-7 cells

The analysis of cellular functional tests revealed an extensive understanding of the cytotoxic and apoptotic pathways induced by *Beta Vulgaris* extracts on MCF-7 cells in contrast to the reference apoptotic inducer, Sts. Important indicators of cellular metabolism, oxidative stress, apoptosis, and membrane damage were assessed, including intracellular ATP level, ROS generation, caspase-3/7 activation, and LDH release, **Table 1**. Sts showed the largest pro-apoptotic effect with 90.41% caspase-3/7 activity, 68.58% LDH release, 81.17% ROS production, and a substantial decline in ATP levels (29.05%). This proved that Sts is a great positive control for triggering apoptosis. Among the beetroot extracts, the BuOH and Ace samples exhibited the most apoptotic activity, with caspase-3/7 activation of 61.48% and 53.72%, respectively. This demonstrated that semi-polar solvents like butanol and acetone effectively eliminated substances that could cause apoptosis, possibly through mitochondrial-mediated processes. Simultaneously, BuOH, Ace, and MeOH extracts showed considerable LDH release (52.78%, 48.34%, and 39.02%, respectively), indicating substantial membrane damage and loss of cell integrity. Furthermore, these extracts raised ROS levels (62.71%, 56.24%, and 48.68%, respectively), suggesting that apoptosis may be influenced by oxidative stress. However, with very little LDH loss, water, EtOAc, and EtOH extracts generated ROS and substantial caspase activation, indicating a less severe cytotoxic effect. There was an inverse relationship between ATP levels and cytotoxicity because the BuOH and Ace extracts dramatically decreased ATP content (48.92% and 54.06%, respectively), which is consistent with metabolic disruption during apoptosis. The water and EtOAc extracts preserved higher ATP levels (88.24% and 73.15%, respectively), indicating less metabolic inhibition and apoptotic activity. These findings demonstrated that the cytotoxic mechanisms of *Beta Vulgaris* were solvent-dependent, with extracts made using semi-polar solvents (BuOH, Ace, and MeOH) having a greater ability to induce apoptosis through increased ROS generation, caspase activation, and mitochondrial malfunction. The results of the anticancer activities were influenced by the rich phytochemical structure of beetroot, particularly betalains, phenolic acids, and alkaloid derivatives. These elements worked together to cause MCF-7 cells to die from oxidative stress. Semi-polar extracts such as BuOH (~51% non-viable) and Ace (~47.5% non-viable) caused more cytotoxicity; however, the water extract maintained a reasonably high viability (~74% survival). This is in agreement with earlier findings by Kapadia et al. (11),, who discovered that beetroot extract, even less effective than doxorubicin, produced cytotoxic effects on MCF-7 cells. In a different study (30), a beetroot extract high in betanin and isobetanin dramatically raised apoptosis-related proteins (Bad, TRAILR4, FAS, p53) in MCF-7 cells and changed the potential of the mitochondrial membrane. A mechanism involving mitochondrial damage and apoptosis is consistent with our findings of enhanced caspase-3/7 activity, increased LDH release, and ROS formation for the BuOH and Ace extracts. The presence of betalains, isoquinoline alkaloids, flavonoids, and cinnamic acids was observed on beetroot peel flour, which also revealed decreases in the viability of the breast cancer cell line MCF-7 (31). This confirms that higher anticancer effects were obtained from semi-polar solvent extracts, which probably contain alkaloids and other non-polar chemical compounds. Variations in the both quantitative and qualitative phytochemical profile obtained with each solvent are responsible for the observed disparities in cell viability and apoptotic response across the extracts. Stronger cytotoxic and pro-apoptotic effects were demonstrated by extracts made with solvents that effectively solubilize bioactive anticancer chemicals, while other extracts showed moderate action because of lower quantities or different classes of compounds. Plant extracts have previously been shown to exhibit solvent-dependent variations in bioactivity, which lend credence to the originality of evaluating various solvent systems to determine which extract best induces apoptosis in MCF-7 cells.

**Table 1:** Effect of beetroot extracts and staurosporine on caspase-3/7 activity, LDH release, ROS levels, and ATP content in MCF-7 cells (%).

	Caspase-3/7 (%)	LDH Release (%)	ROS (%)	ATP (%)
Control	5.14 ± 0.57^g^	4.58 ± 0.41^f^	11.12 ± 0.31^h^	100 ± 3.11^a^
Sts	90.41 ± 3.15^a^	68.58 ± 3.37^a^	81.17 ± 0.15^a^	29.05 ± 1.94^g^
Water	19.25 ± 2.79^f^	17.14 ± 2.82^e^	24.05 ± 1.44^g^	88.24 ± 2.26^b^
EtOH	44.18 ± 3.16^d^	33.69 ± 3.19^d^	43.25 ± 2.58^e^	64.21 ± 2.92^d^
MeOH	49.45 ± 3.14^c^	39.02 ± 2.71^c^	48.68 ± 3.45^d^	65.76 ± 3.62^d^
Ace	53.72 ± 2.49^c^	48.34 ± 3.13^b^	56.24 ± 2.37^c^	57.75 ± 3.69^e^
EtOAc	34.05 ± 3.27^e^	31.54 ± 2.81^d^	38.1 ± 2.78^f^	76.87 ± 3.72^c^
BuOH	61.48 ± 3.08^b^	52.78 ± 2.76^b^	62.71 ± 3.16^b^	48.92 ± 3.61^f^

^*Different letters (a, b, c, d, e, f, g and h) mean significant differences among extracts.^

### Effects of *Beta vulgaris* extracts on protein in MCF-7 cells

**Figure 5:**
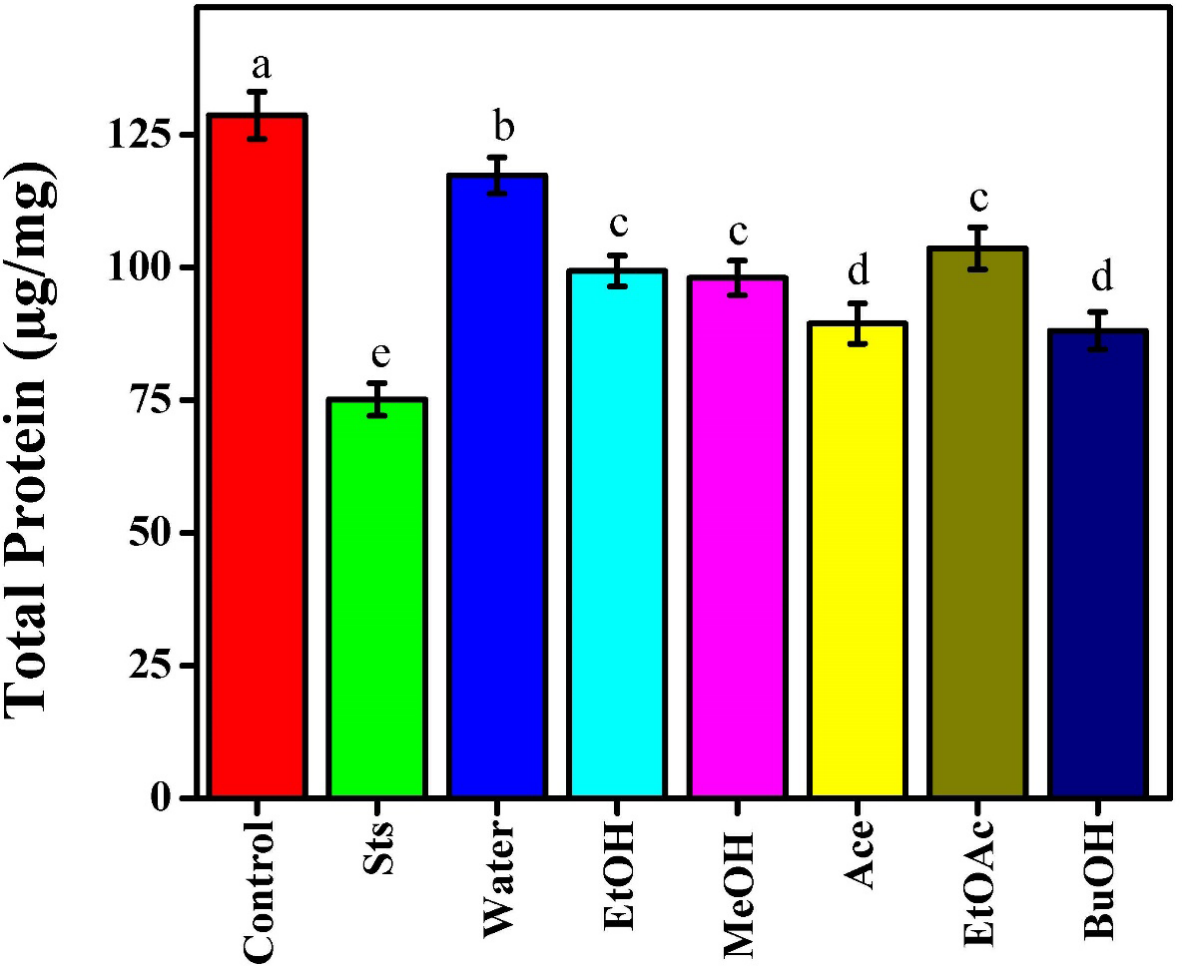
Effect of beetroot extracts and staurosporine on the total protein of MCF-7 cells; as known Different letters (a, b, c, d, e, f, g, and h) mean significant differences among extracts.

Reductions in total protein concentration frequently indicate cytotoxicity, apoptosis, and metabolic inhibition. Total protein concentration variations are indicative of biosynthetic activity and cellular integrity. Sts demonstrated its strong cytotoxic and apoptotic effect by causing the greatest decrease in total protein levels (75.14 µg/mg) as compared to the untreated control (128.65 µg/mg), **Figure 5**. All of the beetroot extracts showed significant solvent-dependent variance. The BuOH (88.07 µg/mg) and Ace (89.42 µg/mg) samples reduced total protein the greatest, indicating considerable suppression of protein synthesis or rapid protein breakdown, in accordance with their previously reported strong caspase-3/7 activity and LDH release. The MeOH (98.02 µg/mg) and EtOH (99.32 µg/mg) extracts showed moderate reductions, indicating partial cytotoxic effects that can be connected to the presence of bioactive phenolics and flavonoids that could induce apoptosis. Higher protein levels were maintained in extracts of water (117.31 µg/mg) and EtOAc (103.58 µg/mg), indicating decreased toxicity and improved cell survival. This outcome was in line with earlier experiments that demonstrated these extracts had less of an effect on caspase activation and ATP depletion. The results demonstrated that the amount of protein in cells was more affected by extracts prepared with somewhat polar solvents, particularly BuOH, Ace, and MeOH. This is most likely due to the extracts’ inclusion of compounds that inhibit protein synthesis and metabolic activities. Nowacki et al. (30), reported similar results, showing that betanin-rich *Beta Vulgaris* extracts caused apoptosis in MCF-7 cells by suppressing protein synthesis and causing mitochondrial malfunction. Similarly, Kapadia et al. (11), found that exposure to beetroot extract resulted in decreases in cellular proteins and viability, corroborating that phytochemicals, including betalains, alkaloids, and phenolic acids, are important in mediating cytotoxicity. Protein then triggers apoptosis by activating caspase 9 and releasing mitochondrial cytochrome c. The morphological and biochemical alterations of apoptosis are caused by the activation of caspase 9, which in turn triggers the activation of caspase 3 and the destruction of several intracellular proteins (18, 32).

### Effects of *Beta vulgaris* extracts on oxidative stress and nitric oxide production

**Figure 6:**
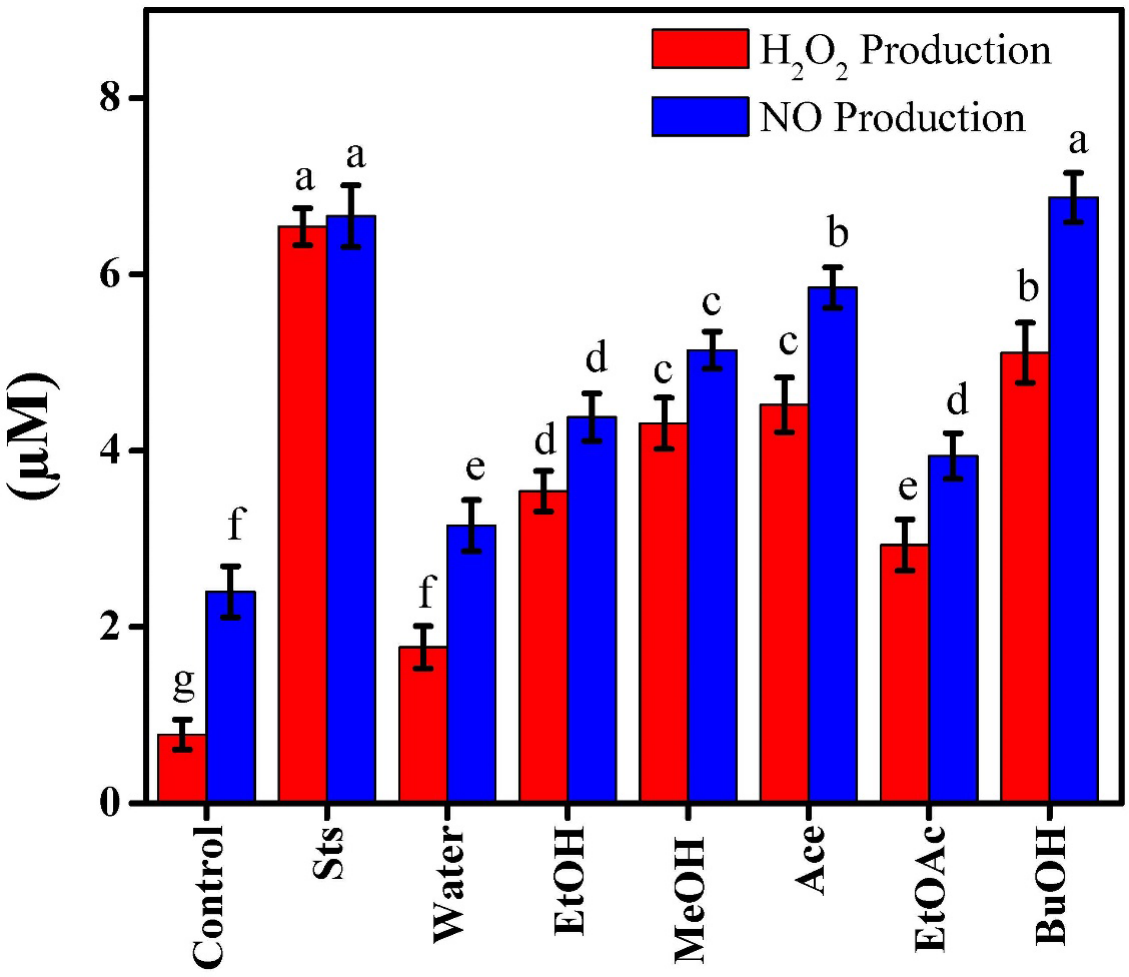
Effect of beetroot extracts and staurosporine on the oxidative stress and nitric oxide production of MCF-7 cells; as known Different letters (a, b, c, d, e, f, g, and h) mean significant differences among extracts.

ROS, or H_2_O_2_, are spontaneously generated during cellular metabolism. They function as a signaling molecule at low concentrations and induce oxidative stress at high concentrations, which damages lipids, proteins, and DNA and initiates apoptosis. Elevated H_2_O_2_ frequently denotes cytotoxicity and activation of pro-apoptotic pathways in cancer research (17). NO is produced by NOS enzymes, such as constitutive nitric oxide synthase (cNOS) and inducible nitric oxide synthase (iNOS). While iNOS can produce larger amounts of NO when activated by inflammatory cytokines, contributing to vascular microcirculation disruptions and gastric mucosal injury, cNOS can continuously release low NO levels within the physiological standard level (3). H_2_O_2_ and NO levels were measured in order to investigate the impact of red beetroot extracts on oxidative stress and NO generation in MCF-7 cells. In comparison to the untreated control (0.78 µM H_2_O_2_ and 2.4 µM NO), **Figure 6**. The positive control, Sts, enhanced both H_2_O_2_ (6.54 µM) and NO production (6.66 µM), indicating the activation of oxidative stress and pro-inflammatory signaling in apoptotic cells. BuOH showed the largest rise in oxidative markers (H_2_O_2_: 5.11 µM; NO: 6.87 µM) among the beetroot extracts, followed by Ace (H_2_O_2_: 4.52 µM; NO: 5.85 µM) and MeOH (H_2_O_2_: 4.31 µM; NO: 5.14 µM), suggesting a pro-oxidant activity of the more lipophilic fractions. EtOH (H_2_O_2_: 3.54 µM; NO: 4.38 µM) showed intermediate activity, while water (H_2_O_2_: 1.77 µM; NO: 3.15 µM) and EtOAc (H_2_O_2_: 2.93 µM; NO: 3.94 µM) extracts showed moderate increases. According to Lechner and Stoner (33), beetroot betacyanins increased ROS and NO levels in tumor cells, encouraging apoptosis.

The results indicated that the pro-oxidant activity of beetroot extracts contributed to the cytotoxic effects in MCF-7 cells. Variations in the phytochemical composition, such as betacyanins and other phenolic compounds, which were more concentrated in the BuOH and Ace extracts, may be the cause of the varied activity between extracts. In line with earlier research on beetroot’s anticancer potential, these results generally confirmed that red beetroot extracts cause oxidative stress-mediated apoptosis in cancer cells (34).

### Effects of *Beta vulgaris* extracts on early and late apoptosis analysis in MCF-7 cells

**Figure 7:**
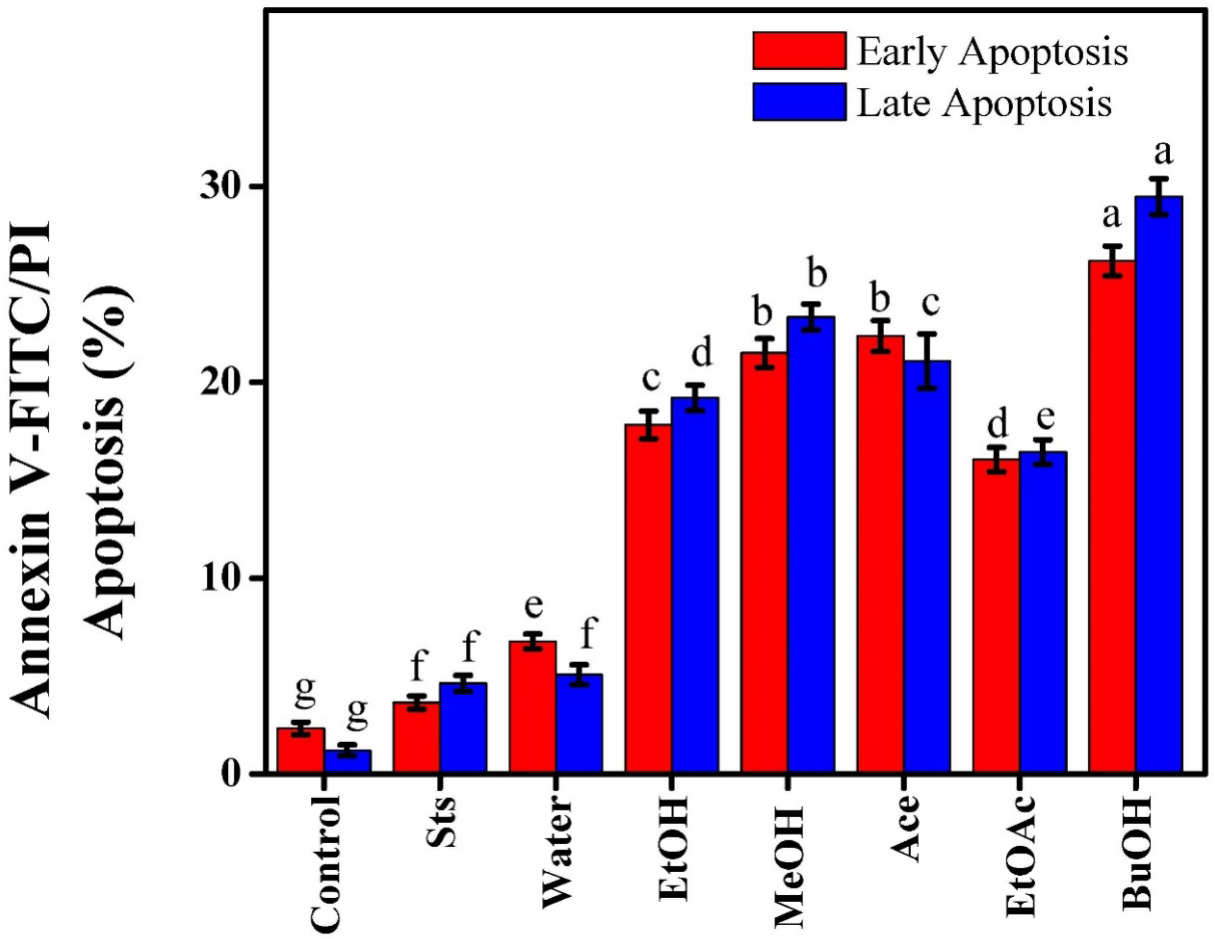
Effect of beetroot extracts and staurosporine on the early and late apoptosis analysis of MCF-7 cells; as known Different letters (a, b, c, d, e, f, g, and h) mean significant differences among extracts.

Early apoptosis is characterized by loss of cell membrane asymmetry and translocation of phosphatidylserine from the inner to outside regions. As a result, Annexin V binds to phosphatidylserine (PS) rather than phospholipids (35). PS, a cell membrane component, can frequently be identified in the inner leaflet region. PS is extruded to the membrane’s outer leaflet early in the apoptotic process. Annexin-V is a calcium-dependent phospholipid-binding protein with strong affinity for PS. Flow cytometry can identify annexin-V interaction with PS molecules ejected by early apoptotic cells (36). Annexin V binding to phosphatidylserine indicates early apoptosis, while late apoptosis or necrosis is indicated by both binding to phosphatidylserine and staining of DNA with propidium iodide due to membrane integrity loss (18). Following treatment with red beetroot extracts and Sts, the rates of early and late apoptosis in MCF-7 cells were examined. With 2.33% early and 1.21% late apoptotic cells, the untreated control group showed very little apoptosis, suggesting that the majority of the cells were healthy. While Sts, increased apoptosis, resulting in 3.65% early and 4.63% late apoptotic cells, which is consistent with its known pro-apoptotic activity. Water caused a mild increase in apoptosis (6.77% early, 5.07% late), but extracts of EtOH (17.82% early, 19.2% late), MeOH (21.49% early, 23.33% late), and Ace (22.36% early, 21.08% late) caused stronger responses **Figure 7**. Among the extracts, BuOH had the greatest apoptosis (26.19% early, 29.47% late), while EtOAc had moderate effects (16.05% early, 16.44% late). These findings suggested that bioactive substances that could induce apoptosis in MCF-7 cells were present in more non-polar red beetroot extracts. The results revealed that red beetroot extracts had a solvent-dependent pro-apoptotic effect, with BuOH and Ace extracts acting similarly to Sts. These findings supported previous reports that involved beetroot-derived bioactive chemicals, such as betacyanins and phenolic components, that can trigger intrinsic apoptotic pathways in cancer cells (34).

Chemoprevention relies on activating cell death to prevent cancer from progressing further. Much research using cancer cell lines as an experimental model concentrates on the process of apoptosis. Numerous nutritional studies evaluate natural ingredients to regulate the process. Apoptosis is a beneficial process that helps organisms manage their cell count. Apoptosis and necrosis are distinct processes, yet both eliminate damaged, aberrant, infected, or superfluous cells (1, 37). As the plasma membrane becomes permeable, early apoptotic cancer cells may transition to late apoptotic cells (19).

### Effects of *Beta vulgaris* extracts on cytochrome C release

**Figure 8:**
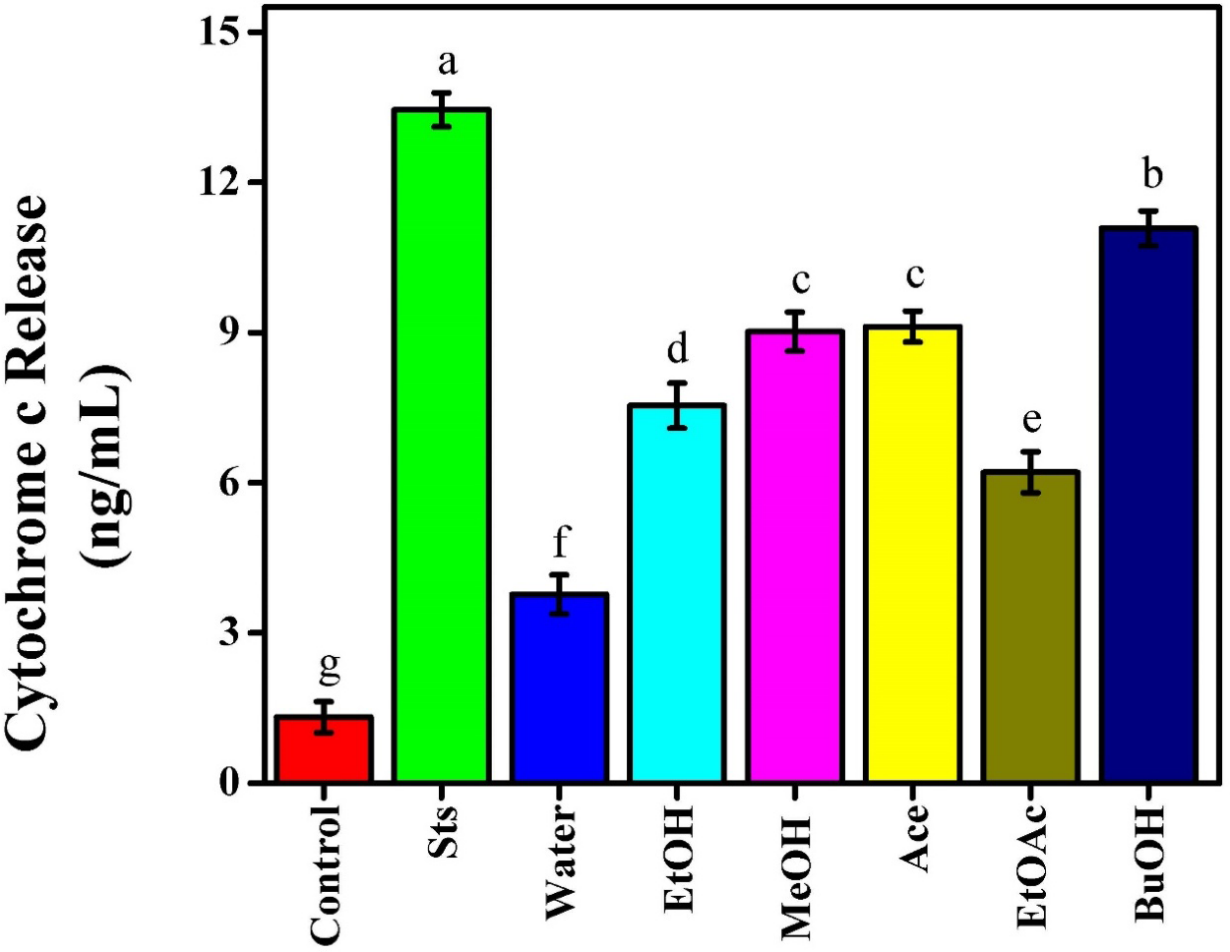
Effect of beetroot extracts and staurosporine on the cytochrome c release of MCF-7 cells; as known Different letters (a, b, c, d, e, f, g, and h) mean significant differences among extracts.

Cytochrome c and caspase-3 are indicators for oxidative damage-induced cell death via the mitochondrial-dependent apoptotic pathway (21). Intrinsic signaling pathways induce apoptosis by generating intracellular signals that directly affect cell targets through mitochondrial-initiated processes when cytochrome c is released into the cytoplasm. In the extrinsic signaling pathway, transmembrane death receptors, including the tumor necrosis factor alpha (TNF-α) receptor, play a crucial role in initiating apoptosis. Extrinsic or intrinsic apoptosis ends with the activation of execution effector caspases, such as caspase-3/6 (38). Caspases activate cytoplasmic endonucleases and proteases to destroy nuclear and cytoskeletal proteins (32). In MCF-7 cells treated with red beetroot extracts and Sts, intrinsic apoptosis was measured by the release of cytochrome c from mitochondria into the cytoplasm. Normal mitochondrial integrity was indicated by the control group’s lowest cytochrome c release (1.32 ng/mL), **Figure 8**. Sts treatment, on the other hand, dramatically increased cytochrome c levels to 13.45 ng/mL, indicating its function as a powerful apoptosis inducer through the mitochondrial pathway. The red beetroot extracts that significantly stimulated mitochondria-mediated apoptosis were BuOH (11.08 ng/mL), Ace (9.12 ng/mL), and MeOH (9.02 ng/mL). The water extract showed the least increase (3.77 ng/mL), whereas the EtOH and EtOAc induced significant release (7.54 ng/mL and 6.21 ng/mL, respectively). These results showed that the more non-polar solvent extracts (BuOH, Ace, and MeOH) included bioactive components that might affect the potential of the mitochondrial membrane and release cytochrome c into the cytoplasm. The results showed that beetroot extracts caused apoptosis through mitochondrial pathways in a solvent-dependent manner, with BuOH exhibiting activity similar to that of Sts. Through the intrinsic signaling pathway, ROS and mitochondria promote apoptosis. ROS release cytochrome c and cause irreversible mortality by oxidizing mitochondrial pores and changing the membrane potential (39). These results were in line with other studies showing that betacyanins and phenolic compounds produced from beetroot induce cytochrome c-mediated caspase activation and mitochondrial dysfunction in cancer cells (11).

### Limitations and future perspectives

The study was limited to an *in vitro* model, which is unable to accurately mimic the intricate systems of a living thing. Furthermore, these results cannot be applied to different subtypes of breast cancer due to the concentration on a single cell line (MCF-7). Furthermore, it was not possible to isolate the precise bioactive substances causing these effects. To fill these gaps, phytochemical characterization and *in vivo* validation should be given top priority in future studies.

## Conclusion

According to the current investigation, red beetroot extracts displayed potent, solvent-dependent cytotoxic and apoptotic effects on MCF-7 cells. As demonstrated by a notable decrease in cell viability and ATP content as well as notable increases in caspase-3/7 activity, cytochrome c release, and apoptosis, butanol exhibited the strongest anticancer potential among the studied extracts. Increased levels of ROS and H_2_O_2_ suggest that oxidative stress contributed to mitochondrial-mediated apoptosis.

## Data Availability

The original contributions presented in the study are included in the article/supplementary material. Further inquiries can be directed to the corresponding author.
